# Conformation and Rheological Properties of Calf-Thymus DNA in Solution

**DOI:** 10.3390/polym8020051

**Published:** 2016-02-11

**Authors:** Lourdes Mónica Bravo-Anaya, Marguerite Rinaudo, Félix Armando Soltero Martínez

**Affiliations:** 1 Grenoble Alpes University, Laboratoire Rhéologie et Procédés (LRP), F-38000, Grenoble 38000, France; monik_ayanami@hotmail.com; 2 Biomaterials applications, 6 rue Lesdiguières, Grenoble 38000, France; 3 Departamento de Ingeniería Química, Universidad de Guadalajara, Blvd. M. García Barragán, Guadalajara C.P. 44430, Jalisco, Mexico; jfasm@hotmail.com

**Keywords:** DNA, hydrodynamic behavior, conformation, electrostatic interactions, temperature

## Abstract

Studies of DNA molecule behavior in aqueous solutions performed through different approaches allow assessment of the solute-solvent interactions and examination of the strong influence of conformation on its physicochemical properties, in the presence of different ionic species and ionic concentrations. Firstly, the conformational behavior of calf-thymus DNA molecules in TE buffer solution is presented as a function of temperature. Secondly, their rheological behavior is discussed, as well as the evidence of the critical concentrations, *i.e.,* the overlap and the entanglement concentrations (C* and C_e_, respectively) from steady state flow and oscillatory dynamic shear experiments. The determination of the viscosity in the Newtonian plateau obtained from flow curves η ($\dot{\gamma }$) allows estimation of the intrinsic viscosity and the specific viscosities at zero shear when C[η] < 40. At end, a generalized master curve is obtained from the variation of the specific viscosity as a function of the overlap parameter C[η]. The variation of the exponent *s* obtained from the power law η~$\dot{\gamma }$^−*s*^ for both flow and dynamic results is discussed in terms of Graessley’s analysis. In the semi-dilute regime with entanglements, a dynamic master curve is obtained as a function of DNA concentration (C_DNA_ > 2.0 mg/mL) and temperature (10 °C < *T* < 40 °C).

## 1. Introduction

DNA in the double helical structure is a long, thin. and locally cylindrical polyelectrolyte chain [1]. DNA results from four different subunits of monomers called deoxyribonucleotides arranged in a precise linear sequence [2,3]. Each deoxyribonucleotide is formed of a nitrogenous base, a deoxyribose sugar, and a phosphate group. DNA is a semi-flexible molecule having a significant possible rotation around a certain number of links in the sugar-phosphate backbone [4]. The importance of this biomolecule relies in the fact that its linear sequence encodes the genetic information [5,6,7]. Its two polymeric strands are wound together to form the double helix of DNA [8]. Before cell division, the two DNA strands are separated, so they operate as a template for the synthesis of a new complementary strand, immediately generating two identical double-helical molecules [9,10].

DNA solutions usually have high viscosities at room temperature (25 °C) and pH = 7.0. Their viscosities decrease when solutions are brought to extreme pH or to temperatures above 70 °C, also depending on the external salt concentration of the solutions [11]. High temperatures and extreme pH cause denaturation (or melting) of double-helical DNA [12,13,14]. DNA denaturation is the process where double-stranded DNA helices unwind and separate into single strands throughout the breaking of hydrogen bonds between bases [15,16]. Each species of DNA is characterized by having a specific denaturation temperature, also referred as melting point (*T*_m_) [17]. DNA melting point increases while increasing the content of guanine–cytosine (G–C) base pairs, since they possess three hydrogen bonds that require more heat energy to dissociate than adenine–thymine (A–T) base pairs [18]. *T*_m_ also depends strongly on the ionic concentration in solution due to the electrostatic screening between phosphate negatively charged sites.

One of the most interesting characteristics of the DNA molecule is that each base pair has two elementary negative charges [5,14,19]. A double-stranded DNA molecule is then characterized by having an exceptionally high linear charge density. Small cations like Na^+^ are usually attracted by DNA negative charges, creating a positively charged cloud around its chain [5]. Several important properties of DNA are strongly dependent on DNA concentration and on the salt content, usually NaCl, due to the electrostatic interactions between negatively charged DNA molecules, and between neighbor phosphate sites on DNA chains and counterions [14,20]. Especially in a diluted solution of polyelectrolyte, such as DNA in water, these electrostatic interchain and intrachain interactions need to be considered. Odijk [21] studied DNA as a wormlike chain characterized by a contour length *L* and a persistence length *l_p_*, bearing charges that interact via electrostatic repulsions increasing the total persistence length. Many authors have extensively studied the effect of electrostatics on the rigidity of the double helix of DNA characterized by its persistence length in dependence of the ionic strength [21,22,23,24,25]. DNA is one the stiffest polymers, with a persistence length of around 50 nm for long chains in 0.1 M aqueous NaCl [22].

Various techniques have contributed to understanding the macroscopic [22,26] and/or microscopic [27] behavior of DNA molecules in solution. In the field of polymers study, there is an important distinction between dilute polymer solutions, where the coiled chains are separated from one another, and more concentrated solutions, where the chains overlap. This threshold is known as the overlap concentration (C*), also considered as a transition region between the dilute regime and the semi-dilute regime [26]. At higher concentrations, it has been reported a second critical concentration, *i.e.*, the entanglement concentration, C_e_ [28,29] or C** [30,31] (depending on the authors) defined as the limit to enter semi-dilute uniformly entangled regime. Several rheological studies of aqueous solutions of DNA have provided information about the dynamics of DNA chains in solution. Raspaud *et al.* [29] and Musti *et al.* [32] published scaling plots of zero shear viscosity as a function of polymer concentration in the semi-dilute with and without entanglements regimes for T2 phage DNA in buffer. They showed that a universal plot could be obtained for all samples when the ratio between the viscosity and the Rouse viscosity (η/η_Rouse_) is plotted as a function of C/C_e_. Over C_e_, the slope of this curve is equal to 3.4. Among several studies of the viscoelastic behavior of calf thymus DNA solutions in buffer, Mason *et al.* [28] reported the dependence of the G modulus plateau (G_0_) and the crossover frequency (ω_c_) with DNA concentration using the following power laws: G_0_~C^2.3^ and ω_c_~C^−2.4^. These values were found to be consistent with the model of entangled semi-flexible polymer coils dissolved in a good solvent [28].

Currently, research has also focused in a more detailed study of the mechanical and dynamical properties of biopolymers study since they directly affect many biological processes, including protein folding and DNA transcription [33,34,35]. As an example, the rigidity of the actin and myosin in muscles provides the structure maintaining the shape of the cell [36], while flexibility of DNA allows to the molecule to undergo a drastic change of state, from an elongated state to a very compact state during the compaction process [19]. Dynamics of biopolymer fluids are a research area of great interest, mainly the flow of DNA in “lab-on-chip” devices, whose applications include the mapping of the genome [37,38]. The study of rheological and flow properties of DNA solutions has much practical importance and can lead to a better understanding of the dynamics of macromolecules [39,40].

In this work, the conformational behavior of calf-thymus DNA in NaCl solutions at different ionic concentrations and the rheological behavior of calf-thymus DNA in Tris–HCl/EDTA buffer are developed. DNA solutions are studied in a wide concentration range, *i.e.*, from the dilute to the semi-dilute with entanglements regimes (0.01 < C_DNA_ < 30 mg/mL), and in the temperature range between 10 and 40 °C. The evidence of two critical concentrations (C* and C_e_) is discussed from steady state and oscillatory dynamic shear experiments. Flow results are analyzed in terms of (1) the viscosity η_0_ in the Newtonian plateau, (2) the critical shear rate $\dot{\gamma }$_c_ for the transition to non-Newtonian behavior, and (3) the exponent from the power law η~$\dot{\gamma }$^−*s*^. The determination of the intrinsic viscosity through the viscosity in the Newtonian plateau allows calculating the molecular weight of calf-thymus DNA. Flow and dynamic measurements data are discussed considering the phase diagram given by Graessley [41] and the two-parameter scaling discussed by Colby *et al.* [42,43].

## 2. Materials and Methods

### 2.1. Materials and Solution Preparation

DNA/Buffer solutions were prepared from samples of calf thymus DNA supplied by Sigma–Aldrich (Toluca, Mexico). A buffer solution was prepared with Tris–HCl and EDTA in order to obtain and maintain pH = 7.3. Trizma, C_4_H_12_ClNO_3_, (Tris–HCl) was used with a purity of 99.0%. Ethylenediaminetetraacetic acid (EDTA), C_10_H_16_N_2_O_8_, was also used with a purity of 99.0%. Appropriate amounts of Tris–HCl (100 mM) and EDTA (10 mM) were used to prepare the buffer solution. After mixing Tris–HCl and EDTA, the pH was checked (pH = 2.75) and then adjusted to pH = 7.3 by adding NaOH solution (3 M). DNA solutions were prepared using appropriate amounts of DNA for each polymer concentration and a solvent consisting of a ratio of 9:1 of HPLC water and Tris–HCl/EDTA buffer (TE buffer). The ionic strength of this TE buffer determined through conductivity measurements was found to be equivalent to 10^−2^ M of NaCl. Sigma–Aldrich Company supplied all reagents. Anhydrous NaCl was used separately to prepare a series of DNA solutions at different ionic concentrations (from 10^−4^ M to 10^−1^ M) to examine the role of ionic concentration on viscosity and conformation. All solutions and dilutions were prepared with HPLC grade water. The vials were closed and sealed with Parafilm^®^ (Bemis NA, Neenah, WI, USA) to prevent water evaporation and changes in the concentration. All solutions were stored in a refrigerator at a temperature of 4 °C in order to prevent degradation.

### 2.2. Purity and Thermal Stability

DNA melting temperatures (*T*_m_) were measured by recording the absorbance A_260_ as a function of temperature (*T*) using a Cary 400 Scan UV–Vis Spectrophotometer. The *T*_m_ cell block contains six cells for samples and six cells for the solvent. Quartz cuvettes were used for all measurements. The solvent cuvettes were filled with water, NaCl solution, or TE buffer solution (depending on the solvent used for the tested sample) and were used as blank. The temperature controlled instrument allows the increase and decrease of temperature with variable increments. In this study, the temperature was raised at a rate of 1 °C/min, from 25 to 90 °C, then it was decreased from 90 to 25 °C at a rate of 5 °C/min.

### 2.3. Rheological Measurements

The rheological behavior of DNA/TE buffer system was studied through flow and dynamic measurements by using DHR-3 and AR-G2 rheometers from TA Instruments Company (New Castle, DE, USA). Three different geometries were used depending on DNA concentration and on the type of experiment carried on: (1) a steel cone with a 60 mm diameter and an angle of 2° was used for DNA solutions with concentrations between 0.01 mg/mL to 0.4 mg/mL (DHR-3 rheometer); (2) a steel cone with a 60 mm diameter and an angle of 1° was used for DNA solutions with concentrations between 0.5 mg/mL to 2 mg/mL (AR-G2 rheometer); (3) a steel cone with a 40 mm diameter and an angle of 2° was used for DNA solutions with concentrations between 2 mg/mL to 30 mg/mL (AR-G2 rheometer).

*Flow experiments:* Steady state measurements were performed in a shear rate range between 1 × 10^−3^ and 1000 s^−1^, using five points per decade. For each DNA sample, each sweep was performed at the following temperatures: 10, 20, 30, and 40 °C, controlled by a Peltier plane.

*Dynamic measurements:* In order to define the linear viscoelastic regime (LVR), oscillation strain sweeps were carried out at an angular frequency of 10 rad/s in a strain range between 0.01% and 100% using 10 points per decade. Frequency sweeps were carried out in the range 0.01 up to 100 rad/s at a selected strain in the LVR, using five points per decade. For each DNA sample, each strain and frequency sweep was performed at the following temperatures: 10, 20, 30, and 40 °C, controlled by a Peltier plane.

## 3. Results and Discussion

### 3.1. Conformation

Absorbance measurements allowed determining DNA concentration in solution. In addition, DNA purity was evaluated by measuring the absorbance from 230 nm to 320 nm to detect other potential contaminants. The ratio A_260_/A_280_ was found to be 1.87 ± 0.11, which is in good agreement with pure DNA ratio reported in the literature (between 1.8 and 2.0) [44]. DNA concentration of the studied samples was determined by measuring the absorbance at 260 nm (A_260_), where DNA absorbs light most strongly [45]. The spectrophotometric measurements at A_260_ can be converted from one absorbance unit at 260 nm to DNA concentration expressed in mg/mL, depending on the nature of the chain [46], *i.e.*, A_260_ = 1 corresponds to 33 μg/mL and to 50 μg/mL for single stranded DNA and double-stranded DNA, respectively. Double-stranded DNA conversion was used for our sample.

**Figure 1:**
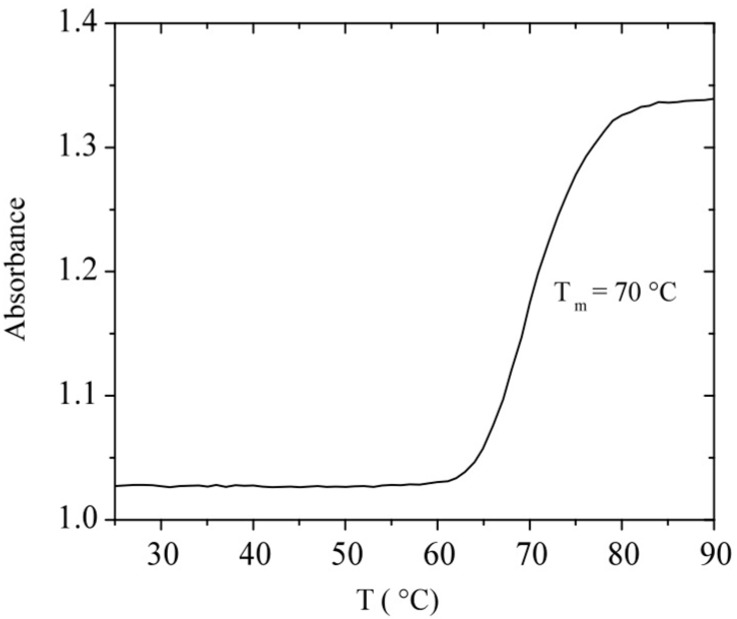
Temperature dependence of the absorbance at 260 nm of calf-thymus DNA (0.042 mg/mL) in TE buffer at pH = 7.3.

Since DNA is a polyelectrolyte bearing a series of phosphate groups along its backbone, it exists in solution under its helical or randomly coiled conformation [47]. It is important to determine its melting temperature, *T*_m_, at the specific conditions used in this research. Figure 1 shows the temperature dependence of the absorbance of calf-thymus DNA at a concentration of 0.042 mg/mL in TE buffer solution. *T*_m_ is determined at the midpoint of the absorbance rise corresponding to *T*_m_ = 70 ± 0.6 °C. This value is in good agreement with Schildkraut *et al.* data for *E. Coli* DNA [12]. We can also observe that up to 55 °C, in our experimental conditions, the double-helical conformation of DNA is preserved. As mentioned before, denaturing conditions such as increase of temperature, increase or decrease of pH, and decrease in Na^+^ ion concentration, disrupt double helix DNA structure, unstack the bases and produce the increase in absorbance [12].

**Figure 2:**
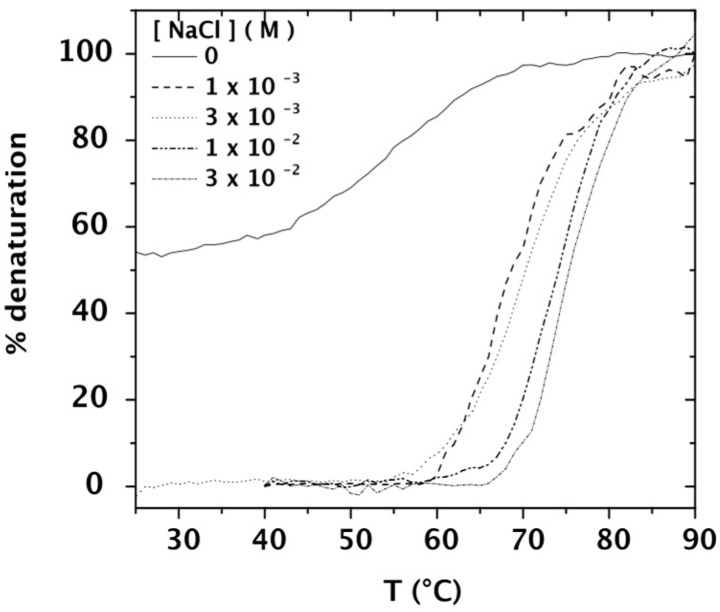
Temperature dependence of the % denaturation calculated from the absorbance at 260 nm of calf-thymus DNA 0.042 mg/mL at salt concentrations up to 3 × 10^−2^ M NaCl, taking as reference the absorbance at 25 °C.

It is well known that the salt concentration influences DNA’s melting temperature [46]. The temperature dependence of the absorbance of calf-thymus DNA at a concentration of 0.042 mg/mL was determined for an external salt concentration ranging between 0 M and 3 × 10^−2^ M NaCl (Figure 2). It is possible to identify that DNA dissolved in water is partially denatured and that presence of salt leads to an increase of DNA melting temperature, as expected [12,19,48]. The observed decrease in *T*_m_ at low salt concentrations is caused by an increase of the electrostatic repulsions between the negative phosphate sites of DNA strands competing with the stabilization by the cooperative hydrogen bonds.

**Figure 3:**
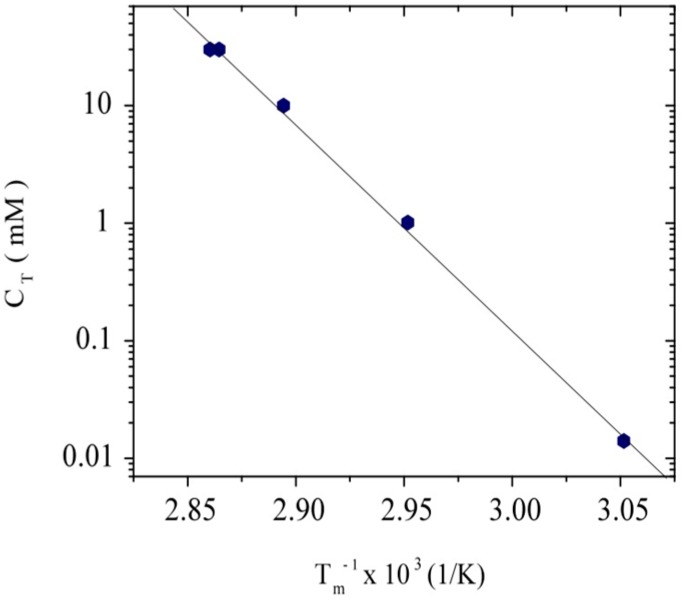
Variation of *T*_m_ with the total ionic concentration for different DNA concentrations and external salt contents.

Figure 3 shows the variation of DNA melting temperature for different DNA and salt concentrations in a semi-log plot. A linear behavior is observed when the total ionic concentration (*C*_T_) is plotted in semi-log as a function of the inverse of *T*_m_, as usually adopted in the literature [18]. In this representation, *C*_T_ includes the contribution of free counterions from DNA and ions from the external salt addition [48].

From our experimental results, agreeing with the literature, the main conclusion is that the double helix DNA conformation is preserved in the TE buffer and in DNA solutions with external salt concentrations higher than 10^−3^ M NaCl in the temperature range from 25 to 60 °C.

### 3.2. Intrinsic Viscosity and Molecular Weight

**Figure 4:**
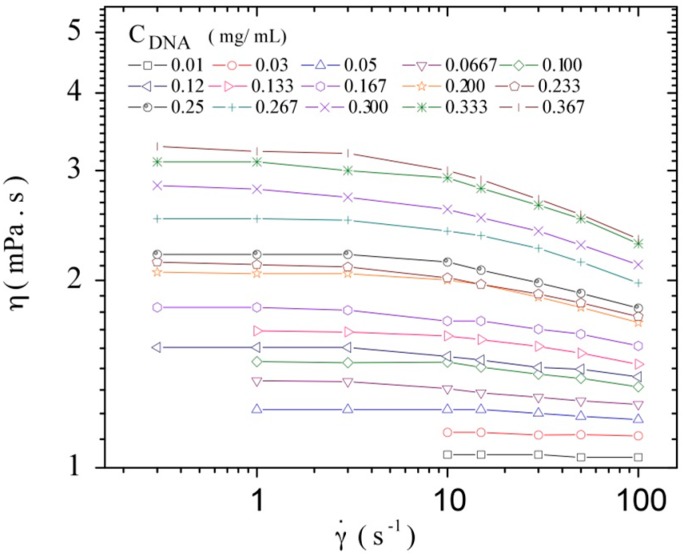
Steady state viscosity as a function of the shear rate for different C_DNA_ at 20 °C in TE buffer.

To obtain the intrinsic viscosity at low shear rate, the cone-plate rheometer DHR-3 was used and DNA shear viscosity was determined in the concentration range between 0.010 and 0.367 mg/mL in TE buffer. A constant viscosity over a range of at least one decade of shear rate was obtained in order to determine with accuracy the zero shear viscosity for each sample tested. Figure 4 shows the measured viscosities at a temperature of 20 °C.

**Figure 5:**
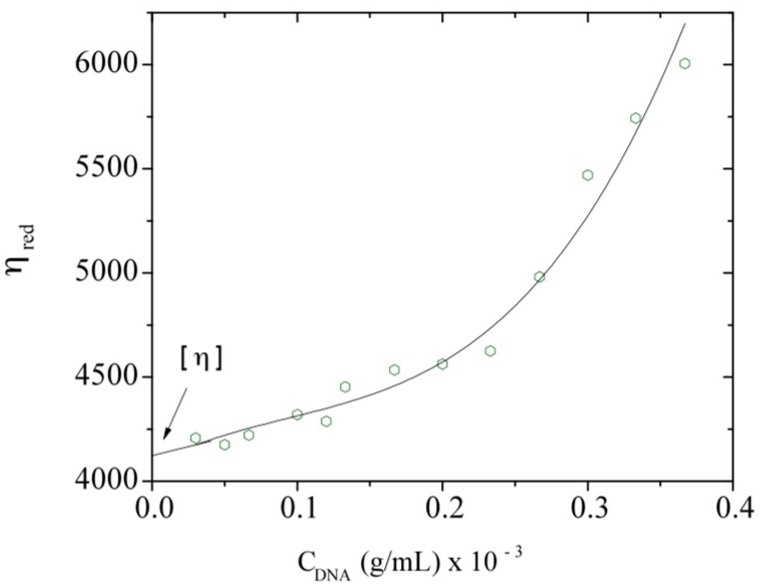
Reduced viscosity, η_red_, dependence with C_DNA_ at a temperature of 20 °C. The solid line represents only a visual aid.

Reduced viscosities were calculated according Equation (1) using the zero shear-rate viscosity from the Newtonian plateau. It was plotted as a function of DNA concentration (C_DNA_) following the Huggins relation (Figure 5):

η_red_ = η_sp_/*C* = [η] + k’ [η]^2^*C* (1)

where η_red_ is the reduced viscosity, η_sp_ is the specific viscosity (equals to (η − η_s_)/η_s_ with η_s_ the solvent viscosity), *C* is the polymer concentration in g/mL, [η] is the intrinsic viscosity in mL/g, and k’ is the Huggins constant.

A deviation from the linear behavior corresponding to the dilute regime is clearly observed at the concentration of 0.23 mg/mL. This concentration could be related to the overlap concentration of the system, C*. The extrapolation to zero concentration gives the intrinsic viscosity, *i.e.*, 4080 mL/g. The viscometric-average molecular weight of the DNA sample can be estimated using the following Mark-Houwink relation (Equation (2)) established by Tsortos *et al.* for a DNA molecular weight range between 2 × 10^6^ and 1.3 × 10^8^ [49]:

[η] = 6.9 × 10^−2^ M^0.70^ (2)

Therefore, the calculated viscometric-average molecular weight for this DNA sample is equal to 6,559,500, which is in the average range of the few reported values for calf-thymus DNA, *i.e.*, between 6,000,000 and 8,000,000 (ultrahigh molecular weight range) [50,51,52]. An interesting example was given by Porsch *et al.* [50] reporting weight–average molecular weight (*M*_w_) for calf-thymus DNA equal to 8,418,000, obtained by size-exclusion chromatography with dual low-angle light scattering/refractometric detection and with an intrinsic viscosity corresponding to [η] = 4850 mL/g.

Concerning the influence of the polymer concentration, Graessley [41] discussed the variation of the chain dimension *R* (with *R*^2^, the chain mean-square end-to-end distance) in a good solvent when polymer concentration increases. At concentrations lower than C*, *R*^2^(C) is independent of C, corresponding to the dilute regime. The overlap concentration is not precisely defined, but it was proposed to correspond to a concentration at which the average spacing between two chains is 2S (0), where S (0) is the radius of gyration of the chain at zero concentration. The radius of gyration and [η] are then two useful characteristics of polymers in dilute solution. The overlap concentration can be estimated as a first approach by using the relation C*~[η]^−1^. In this manner, the calculated value for C* is equal to 0.245 mg/mL, which corresponds to the observed deviation point from the linear dependence of the reduced viscosity with DNA concentration as mentioned before (Figure 5). The first estimation of the radius of gyration may be calculated using C* according to Equation (3) [53]:

C* = [(4/3π <S^2^> ^3/2^N_A_)/M]^−1^ (3)

where N_A_ is Avogadro’s number.

Considering the molecular weight determined by viscosity and Equation (3), an average radius of gyration, <S^2^>^1/2^, equal to 237 nm, is obtained in TE buffer. Then, an approximated value of the persistence length in θ-conditions is calculated for this high molecular weight using the following relation:

<S^2^>^1/2^ = (L*l_p_)/3 (4)

in which *L* is the contour length of the chain and *l_p_* is the intrinsic persistence length found equal to 50 nm. This result is in good agreement with the *l_p_* value usually reported in the literature [22,28].

It is worth mentioning that the entanglements in the semi-dilute regime make the system more complicated and lead to viscoelastic properties that need to be studied by dynamic measurements or analyzed from the effect of shear rate on the viscosity [53].

### 3.3. Rheological Properties of Calf-Thymus DNA

In order to describe the effects of DNA concentration on viscosity and to define the limits between the semi-dilute unentangled and entangled regimes for DNA/buffer solutions, flow and dynamic measurements were carried out in a large range of polymer concentrations. Both series of experiments were performed in TE buffer 9:1 solution at the temperatures of 10, 20, 30, and 40 °C.

#### 3.3.1. Steady Shear Viscometry

In the case of Newtonian fluids, the viscosity usually depends on temperature; however, for polymeric fluids as DNA solutions, the viscosity becomes non Newtonian and depends strongly on shear rate for high MW, on polymer concentration and on the molecular conformation (single strand or double helix). The influence of shear rate on the viscosity of DNA samples was studied in a range of concentration covering mainly the semi-dilute regime to complete the data given in Figure 4. The studies were performed at different temperatures: 10, 20, 30, and 40 °C. These experimental conditions preserve the double helical conformation, as shown from absorbance measurements.

**Figure 6:**
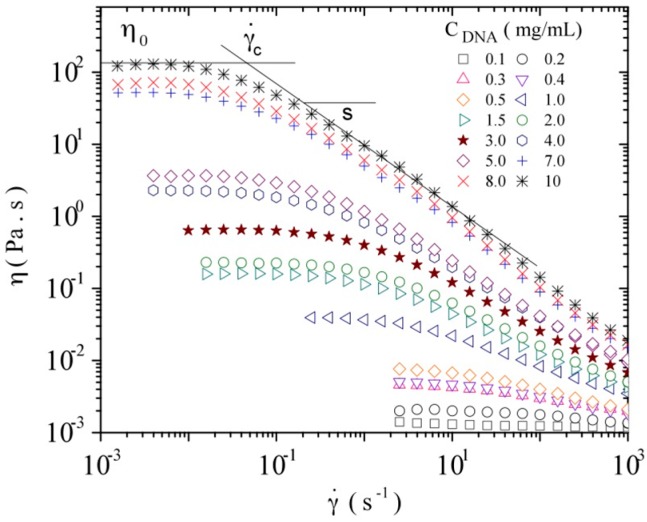
Influence of shear rate on the viscosity of calf-thymus DNA at different DNA concentrations at a temperature of 20 °C in TE buffer.

Data obtained at a temperature of 20 °C are presented in Figure 6. The observed flow curves are characterized by having three important parameters: the viscosity in the Newtonian plateau at low shear rate, η_0_, the critical shear rate, $\dot{\gamma }$_c_, and the slope, *s* [53,54,55].

**Figure 7:**
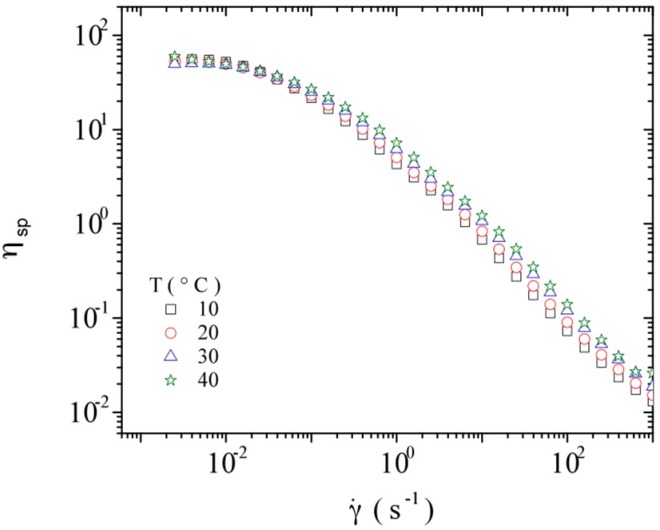
Influence of shear rate on the specific viscosity of calf-thymus DNA at a concentration of 7 mg/mL at the temperatures of 10, 20, 30, and 40 °C.

Figure 7 shows the dependence of the specific viscosity (to take into account the viscosity of the solvent itself) on shear rate for a constant DNA concentration at the different temperatures studied, *i.e.*, 10, 20, 30, and 40 °C. A very small influence of temperature on the specific viscosity of DNA solutions is observed in the studied temperature range, *i.e.*, low influence on the hydrodynamic volume of DNA (reflected by the intrinsic viscosity).

**Figure 8:**
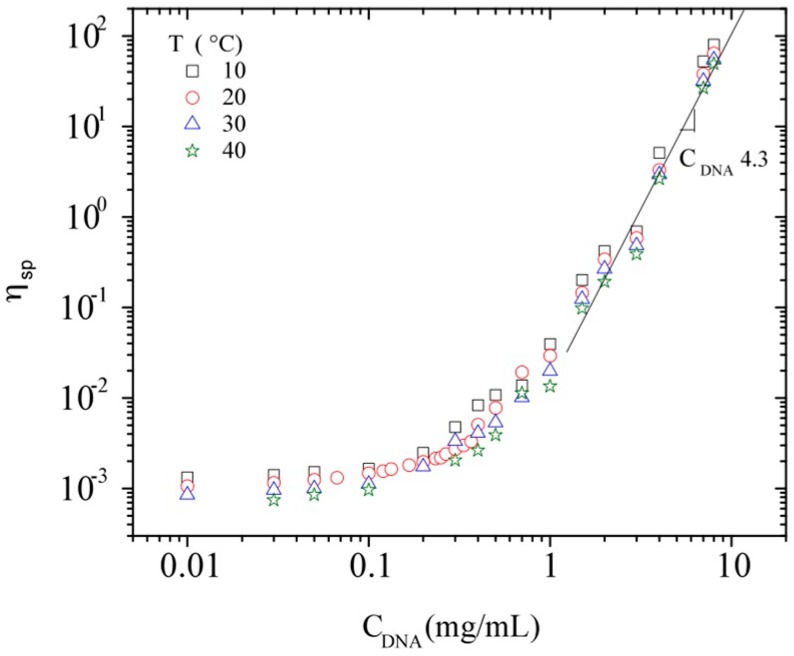
Specific viscosity at zero shear rate as a function of C_DNA_ at 10, 20, 30, and 40 °C.

All the data obtained for the specific viscosity determined in the Newtonian plateau are plotted in Figure 8, in which all the points follow a single curve with a final slope equal to 4.3 at high DNA concentrations. In order to analyze the viscosity variation as a function of polymer concentration and temperature, all experimental values are treated in terms of a master curve. Equation (5) was first used for hyaluronans with various molecular weights [53], from which it was possible to predict any viscosity of polymer solution by replacing *C*[η] in the following expression:

η_sp_ = *C*[η] + k’ (*C*[η])^2^ + B (*C*[η])*^n^* (5)

where k’ corresponds to the Huggins constant and the values *B* and *n* are equal to 7.77 × 10^−3^ and 4.18, respectively. Additionally, a new representation was proposed by Kwei *et al.* [56] (Equation (6)) for the same hyaluronan samples [53,57]:

η_sp_ = *C*[η] [1 + k_1_ (C[η]) + k_2_ (*C*[η])^2^ + k_3_ (*C*[η])^3^] (6)

where the constants k_1_, k_2_, and k_3_ can be calculated as follows: k_1_ = 0.4; k_2_ = (k_1_)^2^/2! = 0.08; k_3_ = (k_1_)^3^/3! = 7.1 × 10^−3^.

**Figure 9:**
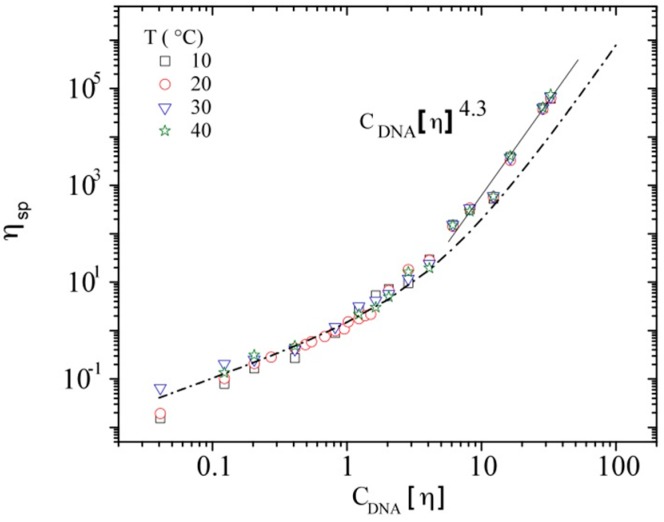
Specific viscosity at zero shear rate as a function of the overlap parameter *C*[η] for calf-thymus DNA solutions in TE buffer pH 7.3 at different C_DNA_ and temperatures (The dashed line represents the master curve expressed by relation 6).

This relation is successfully tested for calf-thymus DNA solutions in the concentration range between 0.01 and 10 mg/mL, using a Huggins constant (identified as k_1_) of 0.4, as found for perfectly soluble polymers (Figure 9). To be able to compare all the results obtained with DNA, but also to compare with other polymers, the majority of the plots are expressed as a function of the overlap parameter C_DNA_[η]. In Figure 9, the specific viscosity at zero shear rate is plotted in a log–log representation as a function of C_DNA_[η].

**Figure 10:**
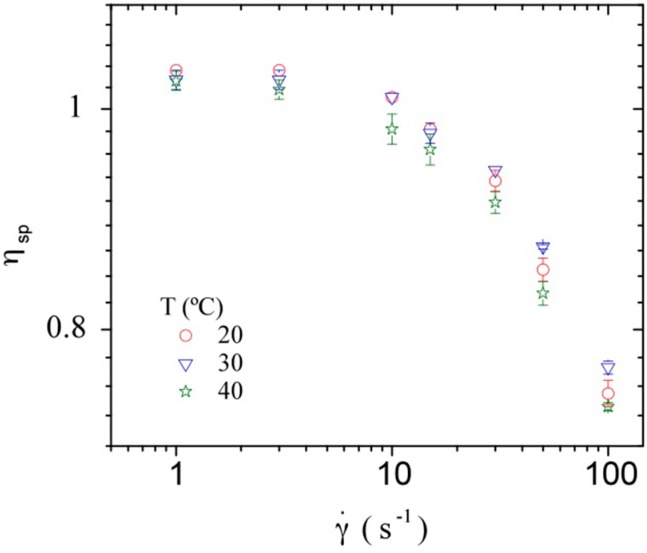
Dependence of the specific viscosity η_sp_, obtained from the shear viscosity at zero shear rate, for a DNA concentration of 0.2 mg/mL in NaCl 1 × 10^−2^ M, on shear rate at the different temperatures studied, *i.e.*, 20, 30 and 40 °C.

The advantage of this simple representation is to be able to calculate η*_sp_* at zero shear rate for any polymeric solution at a given polymer concentration when the intrinsic viscosity is known. A strong deviation from this master curve usually indicates the existence of large aggregates as demonstrated for galactomannan [58]. It is worth mentioning that the value of the intrinsic viscosity determined at 20 °C was adopted for all the temperatures, taking into account that: (1) DNA double helical conformation was identified in the same buffer conditions as used for rheological study, and (2) the specific viscosity is independent of temperature in the range covered (10 to 40 °C) at low polymer concentration (for better sensitivity as shown in Figure 10). The slight influence of temperature on the specific viscosity is attributed to the semi rigid character of DNA. This insignificant role of temperature has been mentioned previously in literature [59].

In addition, the influence of ionic concentration may be considered as playing a role on viscosity when DNA concentration increases. It corresponds to an increase of the total ionic concentration in the solution modifying the electroviscous contribution.

**Figure 11:**
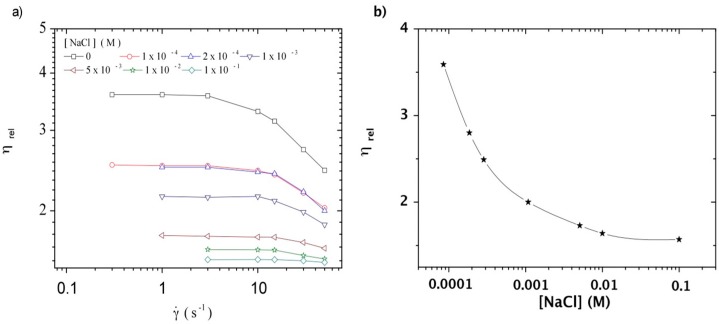
(**a**) Dependence of relative viscosity with the shear rate for a 0.2 mg/L DNA solution in NaCl as solvent at different ionic concentrations. Temperature: 20 °C; (**b**) Dependence of relative viscosity with [NaCl] for a 0.2 mg/mL DNA solution at a temperature of 20 °C.

An example of the role of ionic contribution of DNA on the electrostatic repulsions should be estimated in TE buffer: at 1 mg/mL, the Debye length κ^−1^ ≈ 2.95 nm and for 10 mg/mL it comes that κ^−1^ ≈ 2.7 nm indicating a relatively small influence of the DNA concentration itself. The influence of ionic concentration is evidenced by the viscosity data obtained for one polymer concentration as a function of the shear rate at different concentrations in NaCl as shown in Figure 11a. As usually demonstrated for polyelectrolytes, viscosity at zero shear rate decreases sharply at low ionic concentration. Then, it levels up over 10^−2^ M NaCl (ionic concentration similar to TE buffer) as shown in Figure 11b. At NaCl concentrations higher than 1 × 10^−2^ M, long-range interchain electrostatic interactions between DNA chains in solution are screened and only a small variation of the hydrodynamic volume occurs due to intrachain electrostatic repulsions as usually for semi-rigid polymers.

Taking into account the insignificant influence of temperature and ionic contribution of DNA on its hydrodynamic volume, Figure 9 allows predicting the viscosity for different concentrations and molecular weights of different polymers, as shown by Berriaud *et al.* [60], Milas *et al.* [61], and Fouissac *et al.* [31] for hyaluronans. At low polymer concentration (C[η] ≤ 1) the Huggins relation (Equation (1)) applies, as also recalled by Raspaud *et al.* [29]. These authors found a scaling exponent relating viscosity to hydrodynamic volume (or concentration) equal to 1.3, in agreement with the Rouse model. Equation (6) in the dilute domain should give an average value of 1.2. Then, for the semi-dilute unentangled regime, the viscosity data superpose with the development calculated by Equation (6) with a progressive increase of the slope.

For DNA at the highest concentrations, the concentration C** is obtained when the linear behavior in this curve starts. Mason *et al.* also defined this concentration as the entanglement concentration, *C*_e_ [28]. For the linear domain, the calculated slope is 4.3 (η_sp_~C^4.3^), which presents a slight deviation from the master curve having a maximum slope of 4 (Equation (6)). This kind of deviation was also detected for the reported experimental values for hyaluronan in 0.1 N NaCl (with a slope of 4.18) and xanthan in 0.1 N NaCl (with a slope of 4.24) [30]. The limit for linear behavior in this curve is around C[η]~10, the starting point of semi-diluted entangled domain, *i.e.*, 2.45 mg/mL for DNA. The obtained behavior in the DNA concentration range 2.0 < C_DNA_ < 10 mg/mL was found to be consistent with previous results reported by Mason *et al.* obtained at 25 °C [28]. The width of the semi-dilute domain (unentangled and entangled) is such as *C*_e_~10 C*, as mentioned in the literature [42,62].

The slope in the semi-dilute entangled regime deviates from the master curve representation fitting very well in the lower polymer concentration domains. In fact, this may be connected with the reptation regime admitted over C** (or C_e_) for which η/η_Rouse_ varies as (C/C_e_)^3.4^ [29,32]. In these conditions, it comes that η varies as (C/C_e_)^4.42^. This power law is in good agreement with our data where the slope is 4.3.

**Figure 12:**
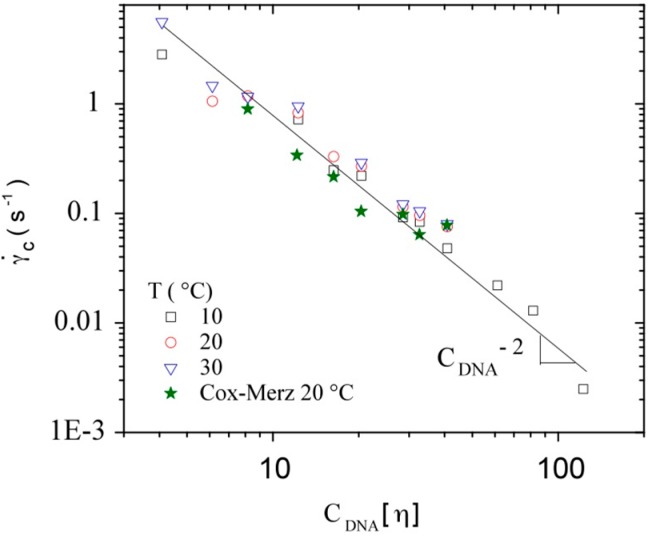
Dependence of the critical shear rate, of the onset of the Non-Newtonian viscosity, with C_DNA_[η] as a function of polymer concentration and for the temperatures 10, 20, and 30 °C. The values obtained by Cox-Merz analysis, discussed later, are also represented.

Considering the transition between Newtonian and non-Newtonian regimes in flow experiments (critical shear rate), Figure 12 is a log-log plot of $\dot{\gamma }$_c_ *versus* C_DNA_[η] for DNA concentrations in the semi-dilute regime at different temperatures. Due to lack of sensitivity, only the semi-dilute domain is covered from 2 mg/mL up to 10 mg/mL. As a small influence of the temperature on the critical shear rate values is found, a single curve is obtained in which all concentrations and temperatures are plotted. When the shear rate increases, the viscosity decrease was attributed to structural changes in the solution such as disentanglements, alignments of the molecules in the flow, or to conformational modifications of the molecules among others. With this curve it is possible to obtain the relation $\dot{\gamma }$_c_~C_DNA_[η]^−2.0 ± 0.1^, also reported for different molecular weight hyaluronans [61]. This exponent is in agreement with the scaling parameter (9/4) corresponding to the longest relaxation time for an entanglement strand in the Rouse theory as mentioned by Colby *et al.* [42]. The calculated values for the critical shear rate with the Rouse model are in good agreement with the experimental values but only for higher concentration (C_DNA_ ≥ 7 mg/mL). The scaling law relates the temperature influence on viscosity with an exponent of 7/12 in a good solvent; it is smaller (considerably low) in our case, probably due to the semi-rigid character of DNA [42].

**Figure 13:**
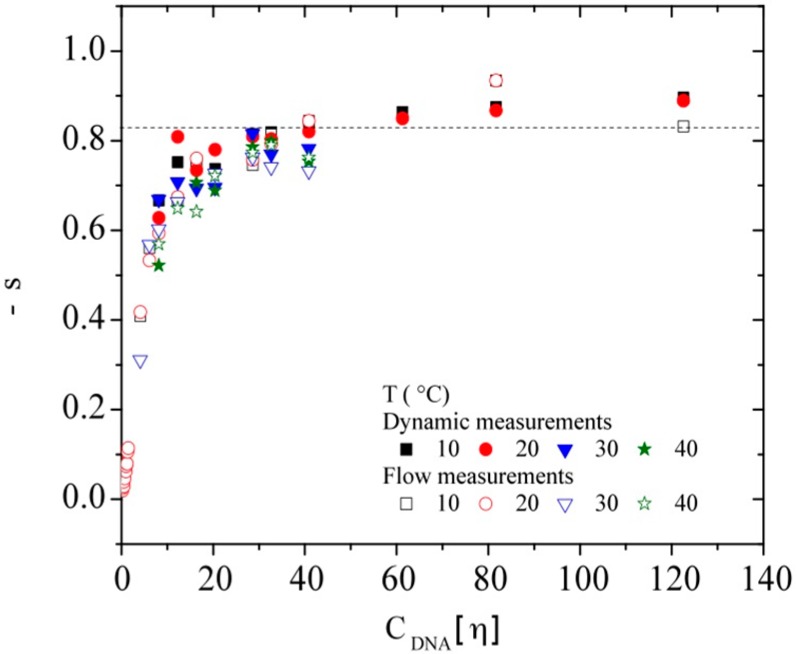
Slope −*s* in the power law region determined on flow curves of calf-thymus DNA in TE buffer at the temperatures of 10, 20, 30, and 40 °C. The values obtained by Cox Merz analysis are also represented. (Dashed line represents the limit proposed by Graessley [63]).

Finally, considering the dependence of viscosity on shear rate, the slope *s* from the power law region of η ($\dot{\gamma }$) as a function of the shear rate was determined at each temperature for each DNA concentration over C*. The obtained results from flow measurements are presented in Figure 13 in terms of –*s* as a function of C_DNA_[η]. The value of –*s* increases and goes to the limit value around −0.82 for a C_DNA_[η] > 35. This limit value agrees with that predicted by Graessley in the entangled regime [63]. The same behavior has also been reported for a wide range of molecular weight hyaluronan samples [60,61] and for xanthan solutions [30].

#### 3.3.2. Dynamic Rheometry

**Figure 14:**
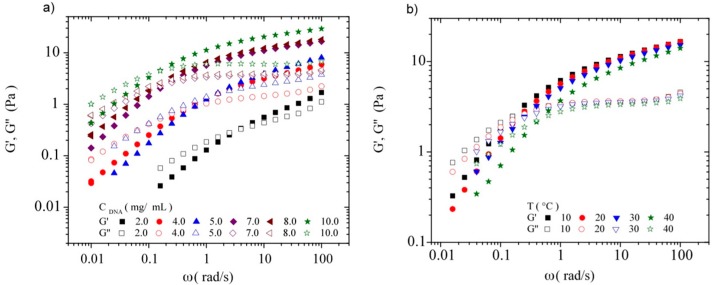
Frequency dependence of the storage (G’) and the loss modulus (G’’) for (**a**) different C_DNA_ in TE buffer at 10 °C and for (**b**) a C_DNA_ of 7 mg/mL in TE buffer at 10, 20, 30, and 40 °C.

By fixing γ = 0.02, inside the linear regime, the spectra for the storage and the loss moduli in the same range of DNA concentrations at the four temperatures were measured subsequently. Figure 14a,b show the dependence of G’ and G’’ with frequency for DNA concentration ranging between 2.0 and 10.0 mg/mL at the temperature of 10 °C and for the DNA concentration 7.0 mg/mL at the temperatures of 10, 20, 30, and 40 °C, respectively. The loss and storage moduli cross at a characteristic frequency (ω_C_) and a modulus value G’ = G’’ = G_c_. The reciprocal of ω_C_ corresponds to the longest relaxation time of the system, τ_c_. This crossover frequency (ω_C_) decreases with increasing DNA concentration and temperature. For solutions at which C_DNA_ ≤ 0.5 mg/mL, the rheological behavior is predominantly viscous at all frequencies (not shown in this figure); however, an elastic behavior is observed at higher DNA concentrations, when the chains start to be entangled.

#### 3.3.3. Rheological Behavior Analysis

**Figure 15:**
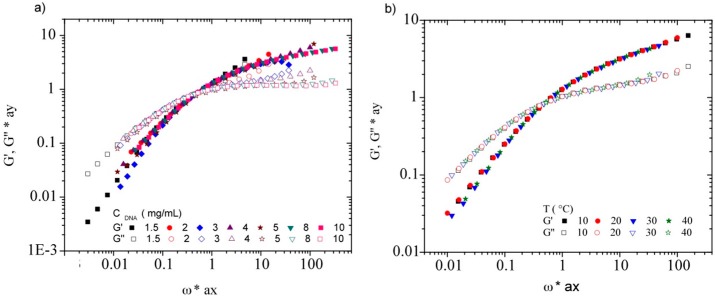
Master curves for the reduced elastic (G’) and viscous (G’’) moduli as a function of the reduced frequency for (**a**) the concentration variation of calf-thymus DNA in TE buffer at a temperature of 20 °C and for (**b**) DNA concentration of 4 mg/mL in TE buffer at the temperatures of 10, 20, 30, and 40 °C.

From dynamic measurements, taking G’(ω) and G’’(ω) at a given DNA concentration as reference it is possible to obtain a master curve as a function of DNA concentration from horizontal (ax) and vertical (ay) translations. First one represents the coefficient of translation of the frequencies and second one the coefficient of translation of the G moduli. Figure 15a shows the master curve obtained for DNA concentration ranging between 1.5 and 10 mg/mL at the temperature of 20 °C, by using polymer concentration C = 4 mg/mL as reference. The same procedure is used for the analysis of the temperature for each DNA concentration. Figure 15 b shows the master curve for a constant DNA concentration at temperatures 10, 20, 30, and 40 °C, using the temperature of 20 °C as reference. Finally, all moduli for DNA concentrations in the concentration range between 1.5 and 30 mg/mL and in the temperature range between 10 and 40 °C collapse in a general dynamic master curve using the frequencies (ax) and the moduli (ay) shifts (Available in Supplementary Material).

**Figure 16:**
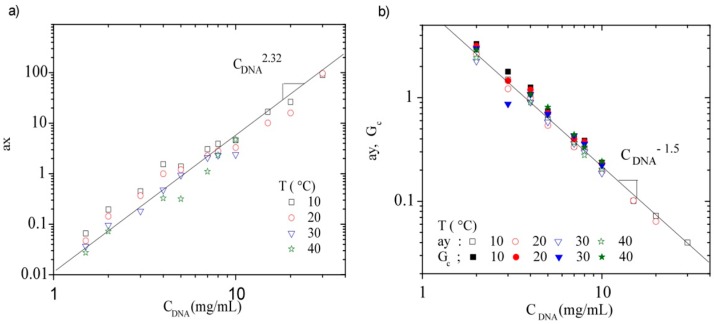
(**a**) Frequency translation coefficient (ax) as a function of DNA concentration and (**b**) G modulus translation coefficient (ay) and G_c_ as a function of DNA concentration, at all studied temperatures.

The frequencies (ax) and the moduli (ay) translation coefficients were plotted as a function of DNA concentration for all temperatures between 10 and 40 °C (Figure 16a,b). These curves were analyzed by fitting the results with a power law giving the following slope values: ax~C_DNA_^2.32 ± 0.16^ and ay~C_DNA_^−1.5 ± 0.05^ respectively. The two series of shifts indicate that the moduli as a function of frequency are mainly imposed by concentration, *i.e.*, density of entanglements. The ax exponent is in good agreement with those previously found on hyaluronan [60]. The Rouse relaxation time for an entanglement strand in the entangled semi-dilute concentration regime varies as C^9/4^, which exponent is in good agreement with the ax shift. Nevertheless, the influence of temperature is lower than predicted by the scaling relation, as observed also on the critical shear rate. This influence causes the dispersion of experimental data observed in Figure 16a. This temperature influence is related to the network relaxation. Concerning ay, G_c_ values superpose with the concentration dependence (Figure 16b). The variation of G_c_, predicted as C^9/4^ [42], is larger than the value obtained on DNA in this work (slope 1.5). This difference could be related with the local stiffness of DNA. It is noted that the temperature dependence is smaller than for ax values and a fortiori than in the scaling relationships proposed for ay (Figure 16b). In this way, ay is mainly imposed by polymer concentration and density of entanglements as ax.

In order to compare our results from flow measurements with those from dynamic measurements, we calculated the complex dynamic viscosity, |η*|, from G’ (ω) and G’’ (ω), as shown by Equation (7):

η* = G*/jω(7)

where G* is the complex modulus given by |G*| = (G’^2^ + G’’^2^)^1/2^

**Figure 17:**
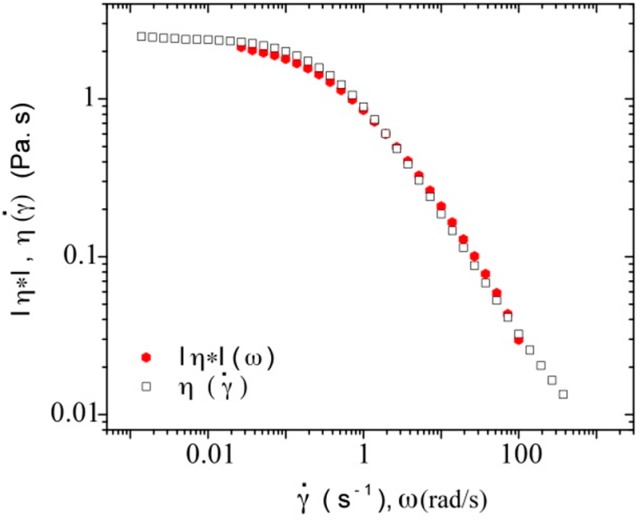
**|**η*|(ω) and η ($\dot{\gamma }$) as a function of shear rate and radial frequency for a DNA concentration of 3.0 mg/mL in TE buffer at a temperature of 20 °C.

The Cox-Merz rule is an empirical relationship in which a correspondence is observed between the steady state shear viscosity, η, plotted against shear rate, $\dot{\gamma }$, and the magnitude of the complex viscosity, |η*|, plotted against angular frequency, ω. This rule has been found to be applicable for many polymer melts and concentrated and semi-dilute solutions. However, some deviations from this rule occur at high frequencies, and the oscillatory data can either over or under estimate the steady state data. Some polymeric systems for which Cox–Merz rule does not apply are cross-linked or gelling systems and most particulate dispersions [64]. Figure 17 presents, as an example, the results obtained for DNA concentration of 3.0 mg/mL in TE buffer at a constant temperature of 20 °C; it shows a good superposition of |η*|(ω) and η ($\dot{\gamma }$) as a function of radial frequency and shear rate, respectively. From these results, it is possible to conclude that there are no strong interchain interactions at least until the DNA concentration of 7 mg/mL at all the studied temperatures.

Then, the characteristic parameters obtained for the slope *s* and for $\dot{\gamma }$_c_ were reported on Figure 12 and Figure 13 respectively. A good agreement is obtained for the experimental values obtained by flow and dynamic measurements indicating that DNA is in good thermodynamic conditions at least up to 7 mg/mL.

## 4. Conclusions

In this paper, the main physicochemical properties of calf thymus DNA in buffer aqueous solution are examined. In a first part, the conformation is characterized as well as its stability using UV–Vis measurements. It is concluded that, in the experimental conditions adopted (TE buffer), the double helical conformation is stable in the range of temperature covered for the rheological study. The melting temperature is found at 70 °C in the buffer used. The influence of the ionic concentration imposed by external salt is also studied. Then, the intrinsic viscosity is determined from the study of viscosity as a function of the shear rate to get the viscosity in the Newtonian plateau. A value of [η] = 4080 mL/g is obtained from which the viscometric-average molecular weight *M*_v_ = 6,559,000 is calculated.

In a second part, the rheological behavior is investigated in a large range of polymer concentration (from 0.01 to 30 mg/mL) in TE buffer and at different temperatures from 10 to 40 °C, where the double helical conformation is stable. For that purpose, flow and dynamic experiments are performed. It is shown that the hydrodynamic behavior is only slightly modified by temperature. We identified three concentration domains: the dilute domain C < C* with C* ≈ 0.23 mg/mL; then, the semi-dilute unentangled regime between C* and C_e_ (or C**) with C_e_ ≈ 2.4 mg/mL followed by the entangled regimes. For the first two regimes, the specific viscosity at zero shear rate follows a development as a function of the overlap parameter C[η]; at larger concentrations, experimental data deviate from this relation and the scaling exponent equals 4.3 in agreement with reptation model. In the semi-dilute regime, the critical value of the shear rate ($\dot{\gamma }$_c_) for the transition from Newtonian to non-Newtonian behavior, as well as the slope of the viscosity as a function of the shear rate (*s*) are examined as a function of the overlap parameter. Dynamic rheology in the linear regime gives a series of curves as a function of DNA concentration and temperatures allowing obtaining a master curve (using a shift using a given reference for C_DNA_ and T). The imposed shifts are analyzed as a function of DNA concentration, the main parameter controlling the solution behavior at constant molecular weight. In the range of concentration covered up to 7 mg/mL, Cox-Merz superposition is valid and all the flow characteristics agree when dynamic complex viscosity and flow viscosity are compared. To conclude, it is necessary to introduce the hypothesis that, up to now, the scaling parameters are introduced for flexible chains (for which the Flory exponent ν = 0.5 in θ-conditions, which is not valid for wormlike chains) and that it is probably needed to reconsider the behavior of semi-rigid chains on certain aspects of the theoretical predictions.

## Supplementary Materials

The following are available online at www.mdpi.com/2073-4360/8/2/51/s1, Figure S1: Master curve for the reduced elastic (G’) and viscous (G’’) moduli as a function of the reduced frequency for the concentration variation of calf-thymus DNA in TE buffer at 10, 20, 30, and 40 °C.

## Abbreviations

The following abbreviations are used in this manuscript:

### Abbreviations

DNA: Deoxyribonucleic Acid
M_w_: weight–average molecular weight
MW: molecular weight
TE: Tris–HCl-EDTA buffer
