# Supplementary material for: Conformation and Rheological Properties of Calf-Thymus DNA in Solution
Source: Polymers (Basel). 2016 Feb 11;8(2):51. doi: 10.3390/polym8020051 (PMC6432584; doi:10.3390/polym8020051)
Supplement: Supplementary file 1 [file polymers-08-00051-s001.pdf]

Supplementary Material: Conformation and Rheological Properties of Calf-Thymus DNA in Solution

Lourdes Mónica Bravo-Anaya, Marguerite Rinaudo, Félix Armando Soltero Martínez

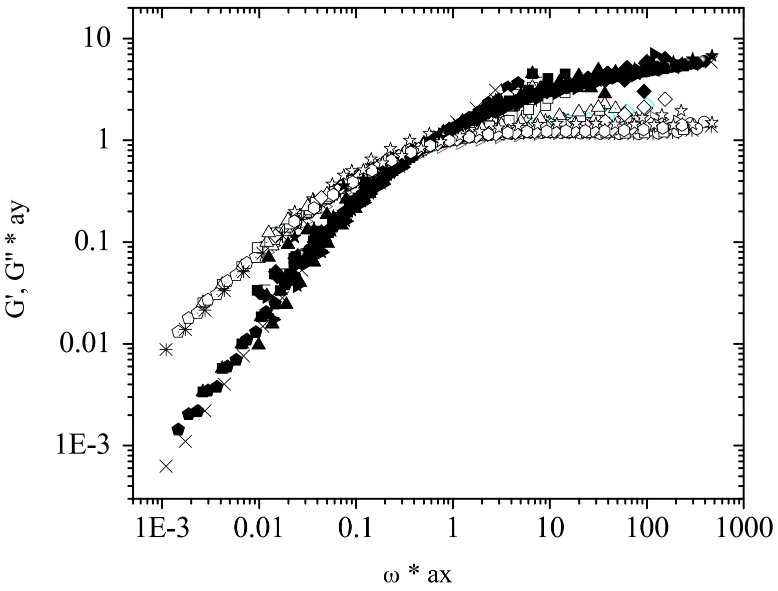

Figure S1. Master curve for the reduced elastic (G′) and viscous (G′′) moduli as a function of the reduced frequency for the concentration variation of calf-thymus DNA in TE buffer at different temperatures (10, 20, 30 and 40 °C).

© 2016 by the authors; licensee MDPI, Basel, Switzerland. This article is an open access article distributed under the terms and conditions of the Creative Commons by Attribution (CC-BY) license (http://creativecommons.org/licenses/by/4.0/).
